# Supplementary material for: The tumor suppressor menin prevents effector CD8 T-cell dysfunction by targeting mTORC1-dependent metabolic activation
Source: Nat Commun. 2018 Aug 17;9:3296. doi: 10.1038/s41467-018-05854-6 (PMC6098065; doi:10.1038/s41467-018-05854-6)
Supplement: Supplementary file 1 — Supplementary Information [file 41467_2018_5854_MOESM1_ESM.pdf]

SUPPLEMENTARY INFORMATIONS

Suzuki et al. The tumor suppressor menin prevents effector CD8 T cell dysfunction by targeting mTORC1-dependent metabolic activation

Supplementary Figure 1

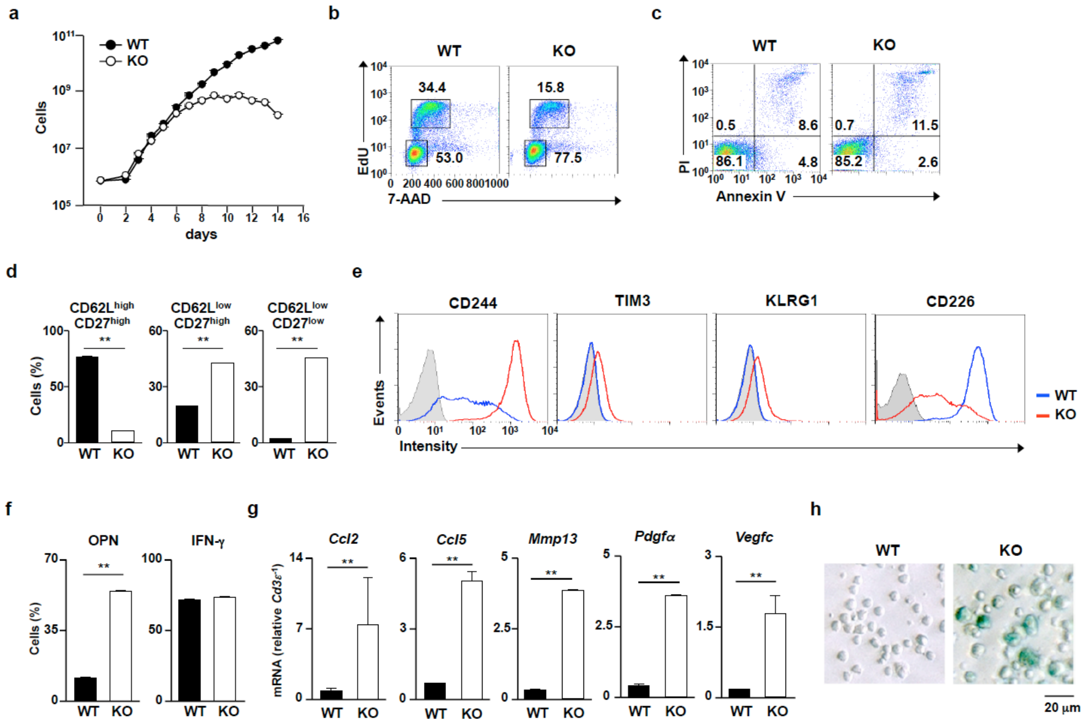

Supplementary Figure 1. Phenotypic characterization of *menin* KO CD8 T cells. Naïve CD8 T cells from the spleen of the WT and *menin* KO mice were stimulated with anti-TCR-β mAb plus anti-CD28 mAb in the presence of IL-2 for 2 days. The cells were then further expanded with IL-2 for indicated days. **(a)** The TCR/CD28-induced proliferation of the *menin* KO naïve CD8 T cells in the presence of IL-2. The average of three independent cultures and standard deviations are shown. **(b)** Representative results of the cell cycle analysis of CD8 T cells from the *menin* KO and WT mice cultured under IL-2 conditions for 7 days. The percentages of G0/G1- and S-phase cells in three independent cultures are shown. **(c)** Representative results of the cell death analysis of CD8 T cells from the WT and *menin* KO mice on day 7. The percentages of cells are indicated in each quadrant. **(d)** The percentages of $CD62L^{high}/CD27^{high}$, $CD62L^{low}/CD27^{high}$, and $CD62L^{low}/CD27^{low}$ CD8 T cells in Figure 1a in three independent cultures with the standard deviation are shown. **(e)** The cell-surface expression of CD244, TIM3, KLRG1 and CD226 on the WT or *menin* KO CD8 T cells on day 7 after the initial stimulation. **(f)** The percentages of Opn- or INF-γ-producing CD8 T cells in Figure 1c in three independent cultures with the standard deviation are

shown. **(g)** The results of the quantitative RT-PCR analysis of mRNA encoding *Ccl2*, *Ccl5*, *Mmp13*, *Pdgfα* and *Vegfc* in the WT or *menin* KO CD8 T cells on day 7. The results are presented relative to the mRNA expression of *Cd3ε* with the standard deviations (n = 3: technical replicates). **(h)** Representative pattern of SA β-Gal staining of the WT or *menin* KO CD8 T cells on day 12. **p< 0.01 (Student's *t*-test).

Supplementary Figure 2

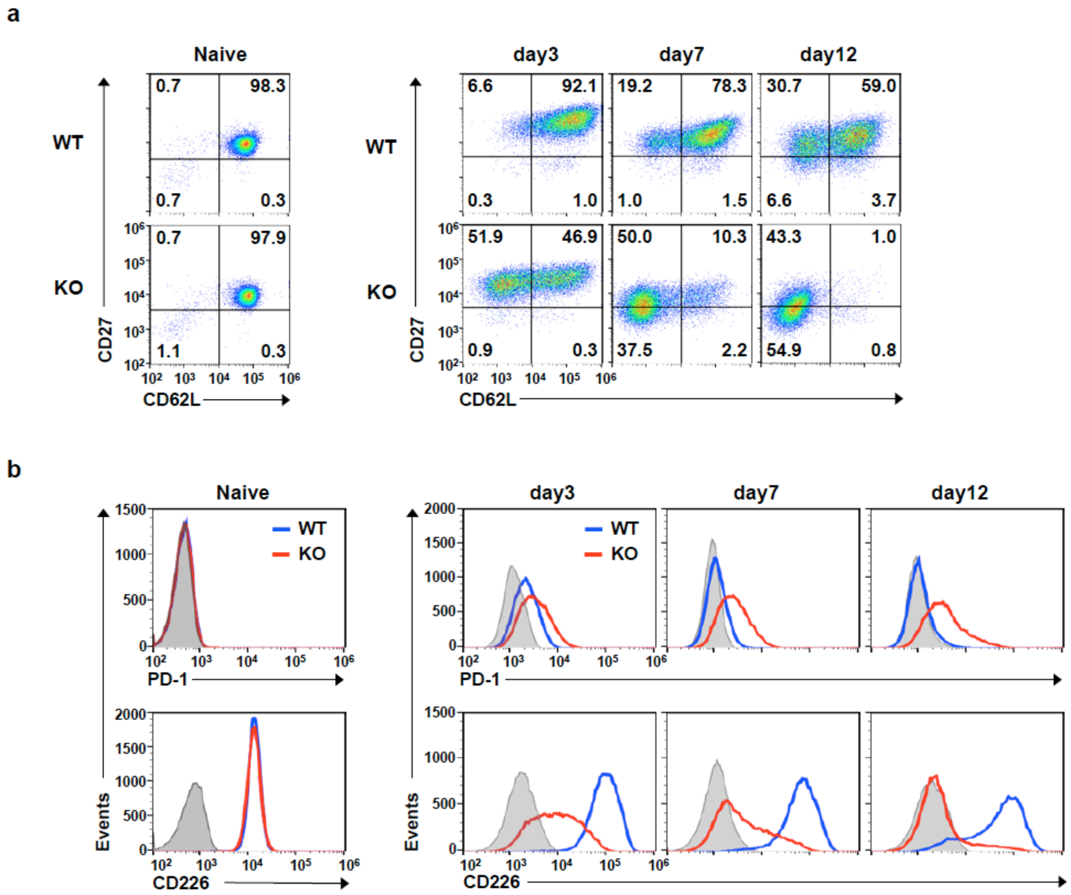

Supplementary Figure 2. The temporal changes of phenotype in *menin* KO CD8 T cells. Naïve CD8 T cells were stimulated with anti-TCR-β mAb plus anti-CD28 mAb in the presence of IL-2 for 2 days. The cells were then further expanded with IL-2 for the indicated number of days. **(a)** Representative FACS profiles of CD62L/CD27 on the cell surface of the WT and *menin* KO CD8 T cells on the indicated days. The percentages of cells are indicated in each quadrant. **(b)** Representative FACS profiles of PD-1 and CD226 on the cell surface of the WT and *menin* KO CD8 T cells on the indicated days.

Supplementary Figure 3

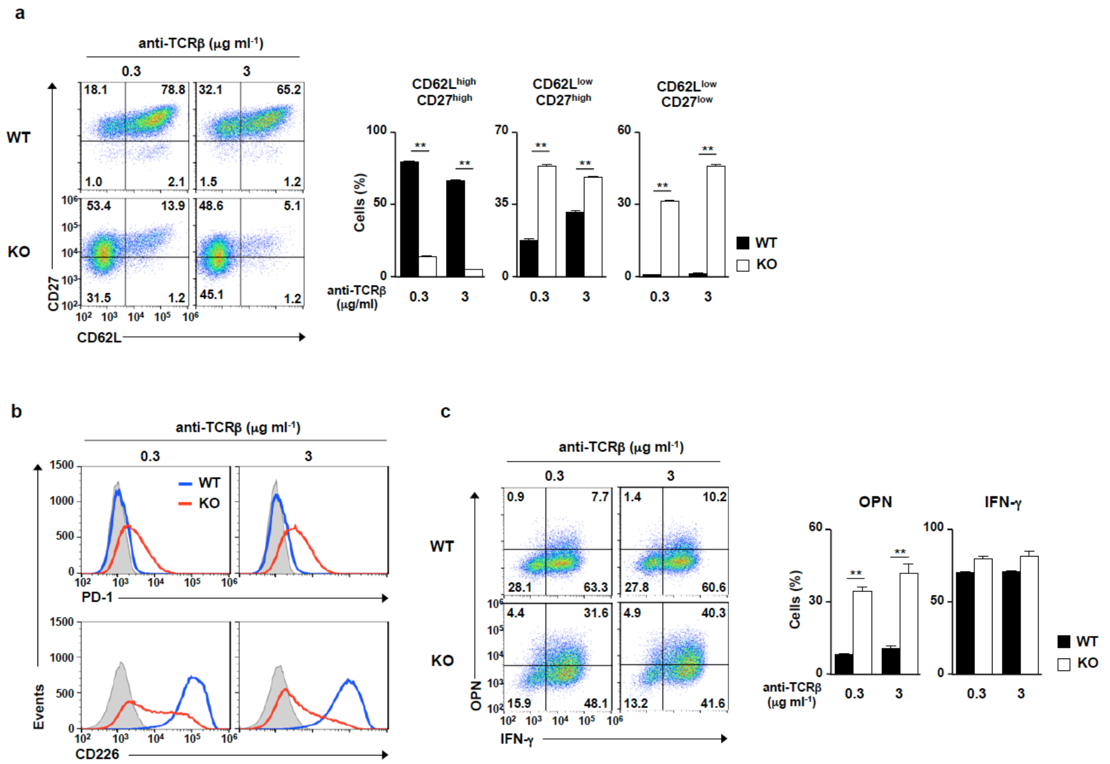

Supplementary Figure 3. Influence of the strength of the TCR-stimulation on phenotype of *menin* KO CD8 T cells. WT and *menin* KO naïve CD8 T cells were stimulated with anti-TCR-β mAb (0.3 or 3 μg ml^{-1}) plus anti-CD28 mAb in the presence of IL-2 for 2 days. The cells were then further expanded with IL-2 for 5 days. An analysis was performed on day 7 after the initial stimulation. **(a)** A representative staining profile of CD62L/CD27 on the cell surface on day 7 (left) and the percentages of $CD62L^{high}/CD27^{high}$, $CD62L^{low}/CD27^{high}$ and $CD62L^{low}/CD27^{low}$ CD8 T cells in three independent cultures with the standard deviation are shown (right). **(b)** The PD-1 and CD226 expression on the cell surface of the WT and *menin* KO CD8 T cells on day 7. **(c)** Representative results of the intracellular FACS analysis of IFN-γ/OPN in WT and *menin* KO CD8 T cells on day 7 (left). The percentages of Opn- or INF-γ-producing WT and *menin* KO CD8 T cells in three independent cultures with the standard deviation are shown (right). **$p<0.01$ (Student's *t*-test).

Supplementary Figure 4

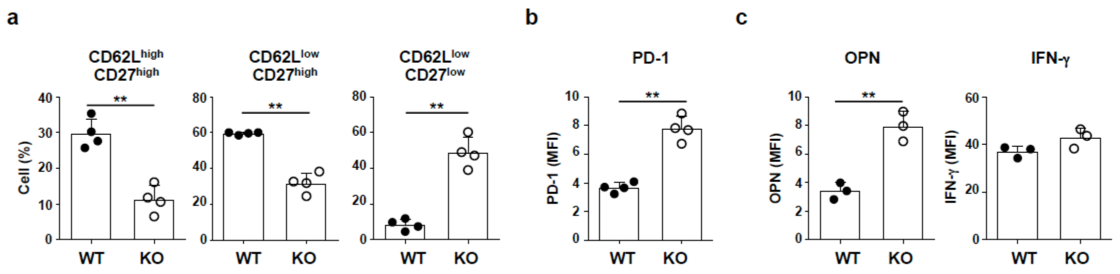

Supplementary Figure 4. Phenotypic characterization of the antigen-specific *menin* KO CD8 T cells after *Lm*-OVA infection. (a) The percentages of CD62L^{high}/CD27^{high}, CD62L^{low}/CD27^{high} and CD62L^{low}/CD27^{low} CD8 T cells in Figure 1g. **(b)** The MFI of PD-1 expression on the cells in Figure 1h. **(c)** The MFI of OPN and IFN-γ expression in the cells in Figure 1i. The results are presented with the standard deviation. (*n*=4 per group). **p<0.01 (Student's *t*-test).

Supplementary Figure 5

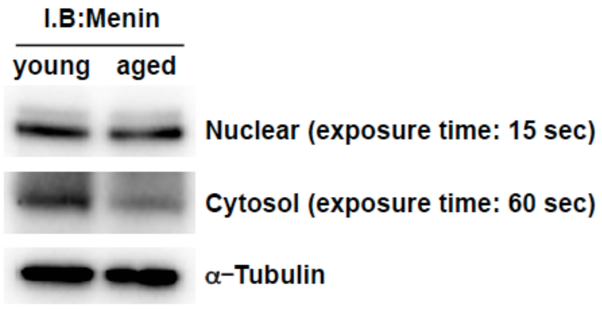

Supplementary Figure 5. The amount of menin protein in aged activated CD8 T cells. The result of the immunoblot analysis of menin in effector CD8 T cells from the young (6 weeks-old) and aged (104 weeks-old) mice. Total CD8 T cells were stimulated with anti-TCR-β mAb plus anti-CD28 mAb with IL-2 for 24 h. The protein amount of menin in the cytosol or nuclear fraction was assessed by immunoblotting. The protein amount of α-tubulin was used as a loading control.

Supplementary Figure 6

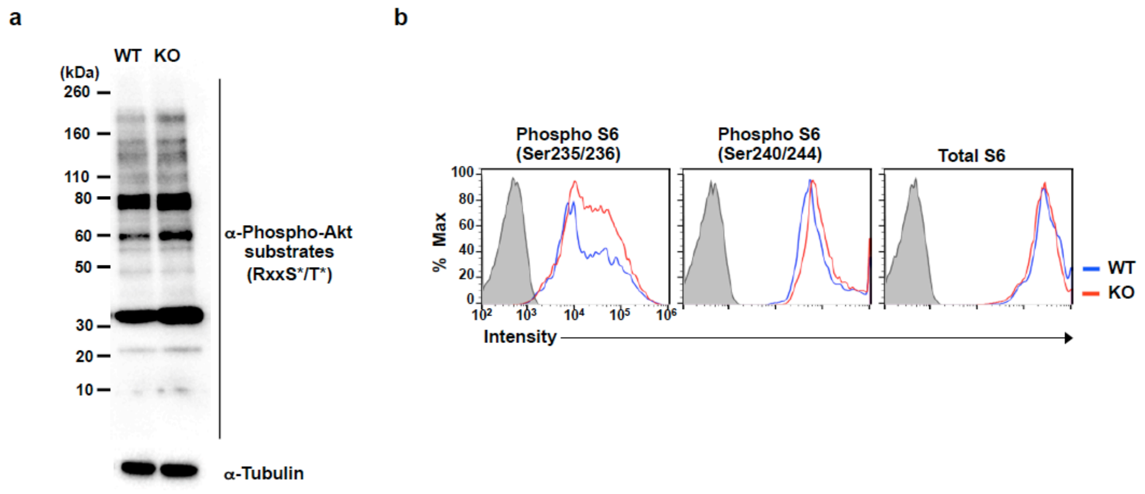

Supplementary Figure 6. The phosphorylation levels of Akt substrates and S6 protein in *menin* KO CD8 T cells. (a) The results of the immunoblot analysis of the phospho-Akt substrates (phospho-RxxS/T) in the WT and *menin* KO activated CD8 T cells. WT or *menin* KO naïve CD8 T cells were stimulated with anti-TCR-β mAb plus anti-CD28 mAb in the presence of IL-2 for 36 h. The protein amount of α-tubulin was used as a loading control. **(b)** Representative FACS profiles of phosphorylated ribosomal S6 (Ser235/236 and Ser240/244) and total ribosomal S6 protein in OVA-specific CD8 T cells. WT and *menin* KO OT1 Tg mice were infected with *Lm*-OVA. OVA-specific activated (CD44-positive) CD8 T cells in the spleen were analyzed 48 h after *Lm*-OVA infection.

Supplementary Figure 7

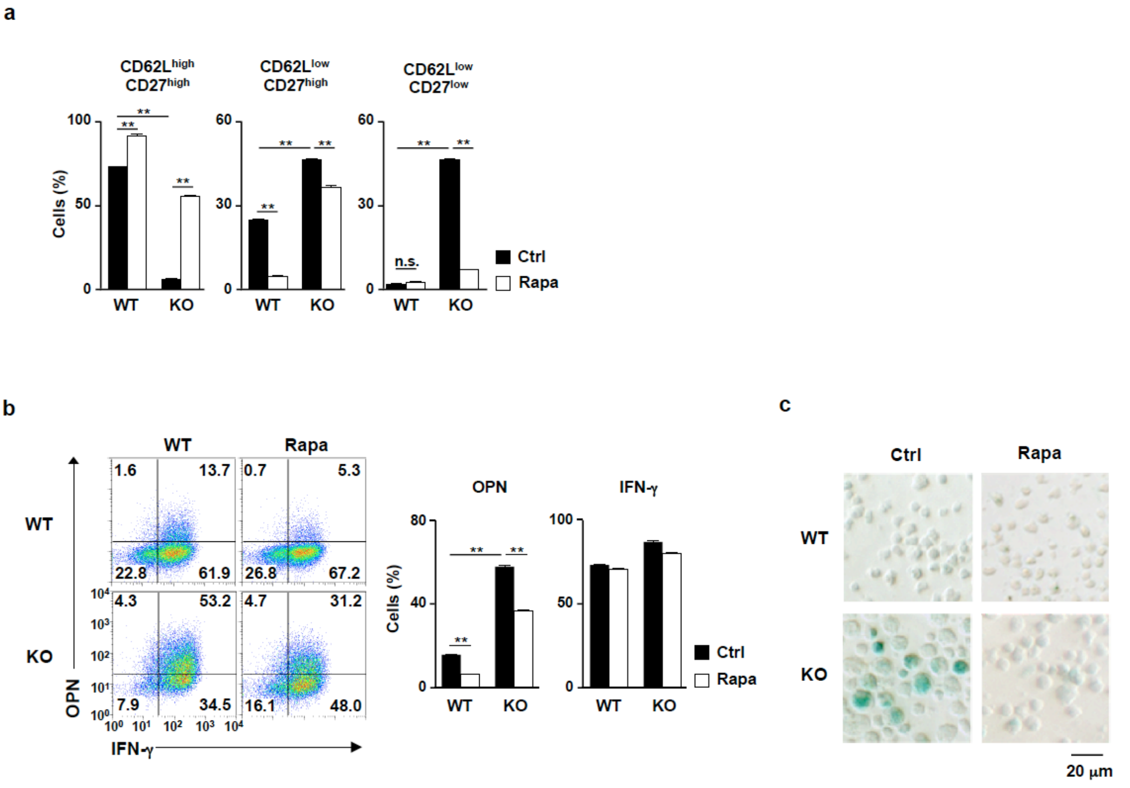

Supplementary Figure 7. The effect of rapamycin on the phenotype of *menin* KO effector CD8 T cells. (a) WT and *menin* KO naïve CD8 T cells stimulated with anti-TCR-β mAb plus anti-CD28 mAb in the presence of IL-2 with or without rapamycin for 2 days, and then cells were cultured with IL-2 in the absence of rapamycin for another 5 days. The percentages of $CD62L^{high}/CD27^{high}$, $CD62L^{low}/CD27^{high}$ and $CD62L^{low}/CD27^{low}$ CD8 T cells in Figure 3a in three independent cultures with the standard deviation are shown. **(b)** A representative intracellular FACS profile of IFN-γ/OPN in the cells in (a) restimulated with anti-TCR-β mAb for 6 h. The percentages of cells are indicated in each quadrant (left). The percentages of OPN- or IFN-γ-producing cells are shown with the standard deviation (n = 3: biological replicates) (right). **(c)** Representative pattern of SA β-Gal staining of the cells in Figure 3e. **p<0.01 (Student's *t*-test).

Supplementary Figure 8

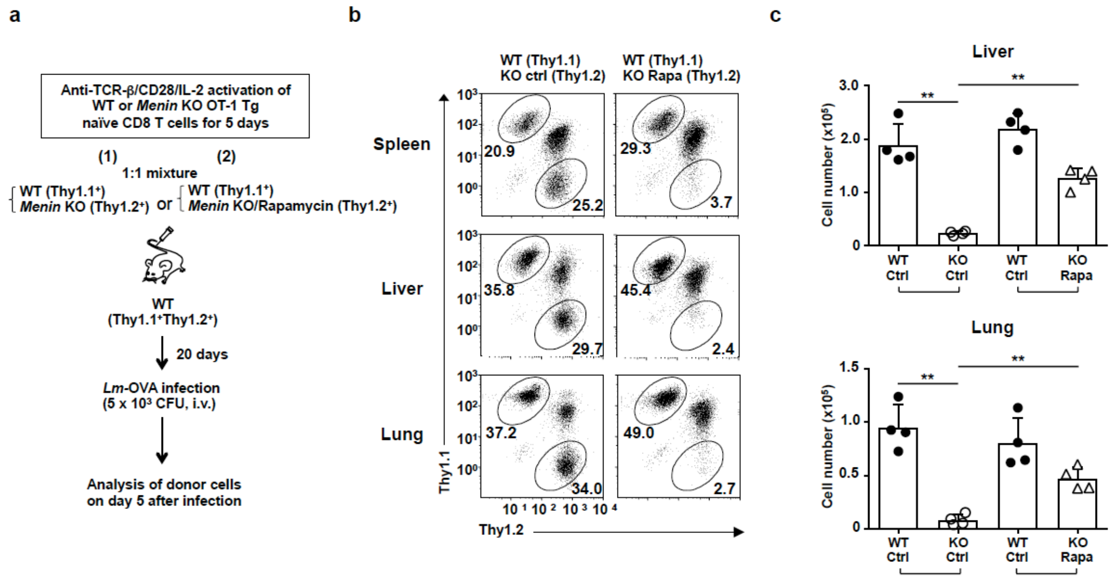

Supplementary Figure 8. The secondary immune response of *menin*-deficient CD8 T cells against Lm-OVA infection. (a) A schematic outline of a competitive assay of the CD8 T cell secondary immune response by adoptive transfer of effector OT1 CD8 T cells into congenic mice. *In vitro*-activated effector CD8 T cells from WT OT1 transgenic (Tg) (Thy1.1^{+}) and *menin* KO OT1 Tg (Thy1.2^{+}) mice were mixed at a 1:1 ratio and adoptively transferred into naïve C57/BL6 mice (Thy1.1^{+} × Thy1.2^{+}). Twenty days after the adoptive transfer, the mice were infected with *Lm*-OVA to activate the donor cells, and the number of OT1 Tg CD8 T cells was assessed. The donor cells from the spleen, liver and lung were analyzed on day 5 after the infection. Representative FACS profiles **(b)** and the absolute number of donor cells (mean ± SD, *n* = 5 per group: biological replicates) **(c)** are shown. **p< 0.01 (Student's *t*-test).

Supplementary Figure 9

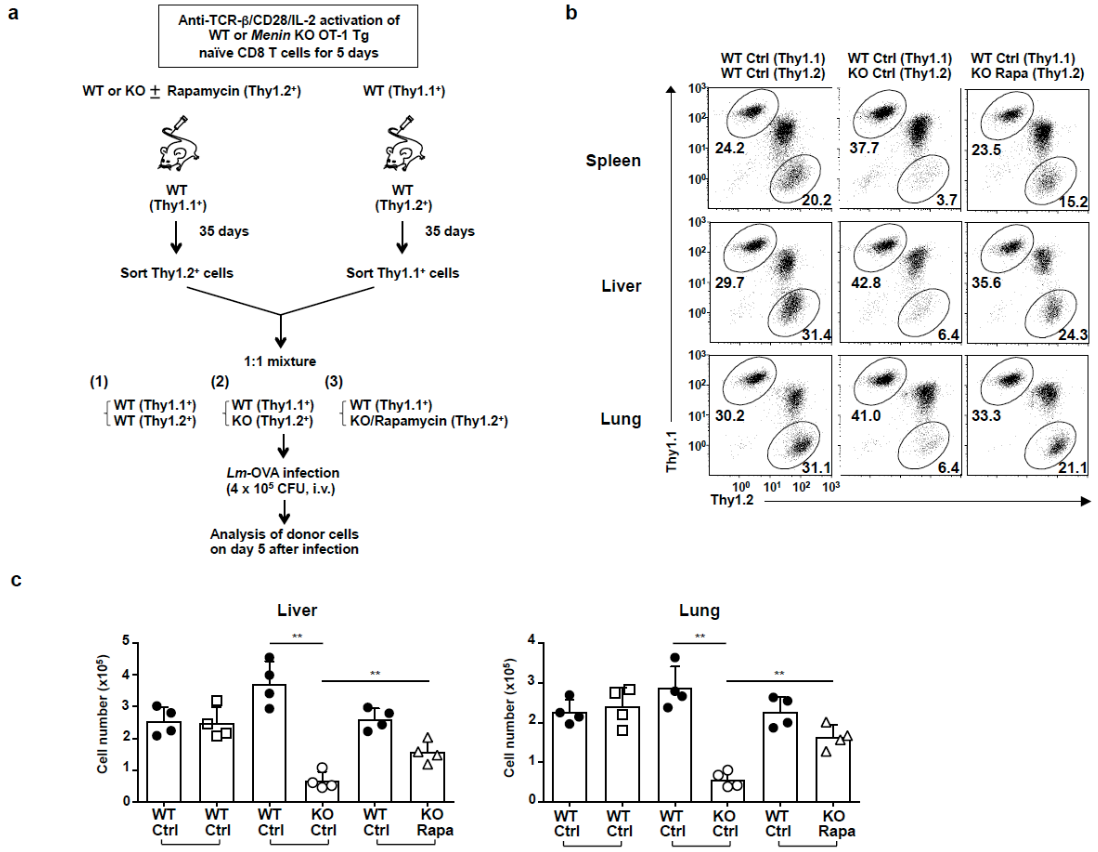

Supplementary Figure 9. The memory response of *menin*-deficient CD8 T cells against Lm-OVA infection. (a) A schematic outline of the competitive assay of the CD8 T cell secondary immune response by the adoptive transfer of memory OT-1 CD8 T cells into congenic mice. WT (Thy1.2$^+$), *menin* KO (Thy1.2$^+$) or rapamycin-treated *menin* KO (Thy1.2$^+$) OT1 Tg effector CD8 T cells were intravenously transferred into WT congenic (Thy1.1$^+$) mice, and WT OT1 Tg effector CD8 T (Thy1.1$^+$) cells were intravenously transferred into WT congenic (Thy1.2$^+$) mice. Thirty-five days after adoptive transfer, the donor cells were recovered from the spleen of the recipient mice, and mixed at a 1:1 ratio (WT: Thy1.1$^+$/WT: Thy1.2$^+$, WT: Thy1.1$^+$/*menin* KO: Thy1.2$^+$ or WT: Thy1.1$^+$/rapamycin-treated *menin* KO: Thy1.2$^+$). The cells were intravenously transferred into naïve C57/BL6 mice (Thy1.1$^+$ Thy1.2$^+$) mice, and then the recipient mice were infected with *Lm*-OVA on the following day to activate the donor cells. The donor cells were collected from the spleen, liver and lung on day 5 after *Lm*-OVA infection. Representative FACS profiles **(b)** and the absolute number of donor cells (mean ± SD, n = 4 per group: biological replicates) **(c)**. **p<0.01 (Student's *t*-test).

Supplementary Figure 10

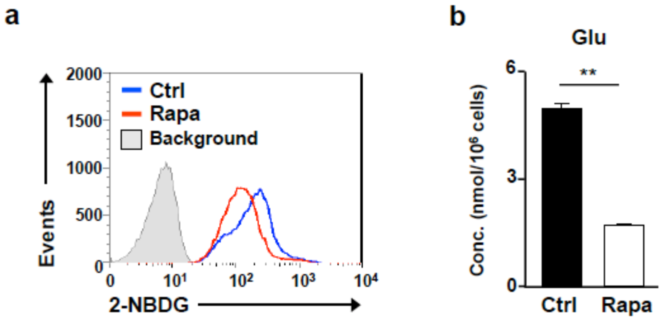

Supplementary Figure 10. Rapamycin inhibits the TCR-mediated activation of glycolysis and glutaminolysis. (a) WT naïve CD8 T cells were stimulated with anti-TCR-β and anti-CD28 mAbs plus IL-2 in the presence or absence of rapamycin (10 nM) for 24 h. 2-NBDG was then added to the cultures for 30 min, and its incorporation was determined by FACS. A representative FACS profile is shown. **(b)** The intracellular level of glutamine stimulated with anti-TCR-β and anti-CD28 mAbs plus IL-2 in the presence or absence of rapamycin (10 nM) for 24 h are indicated (n = 3: biological replicates). The results are presented with the standard deviation. **p<0.01 (Student's *t*-test).

Supplementary Figure 11

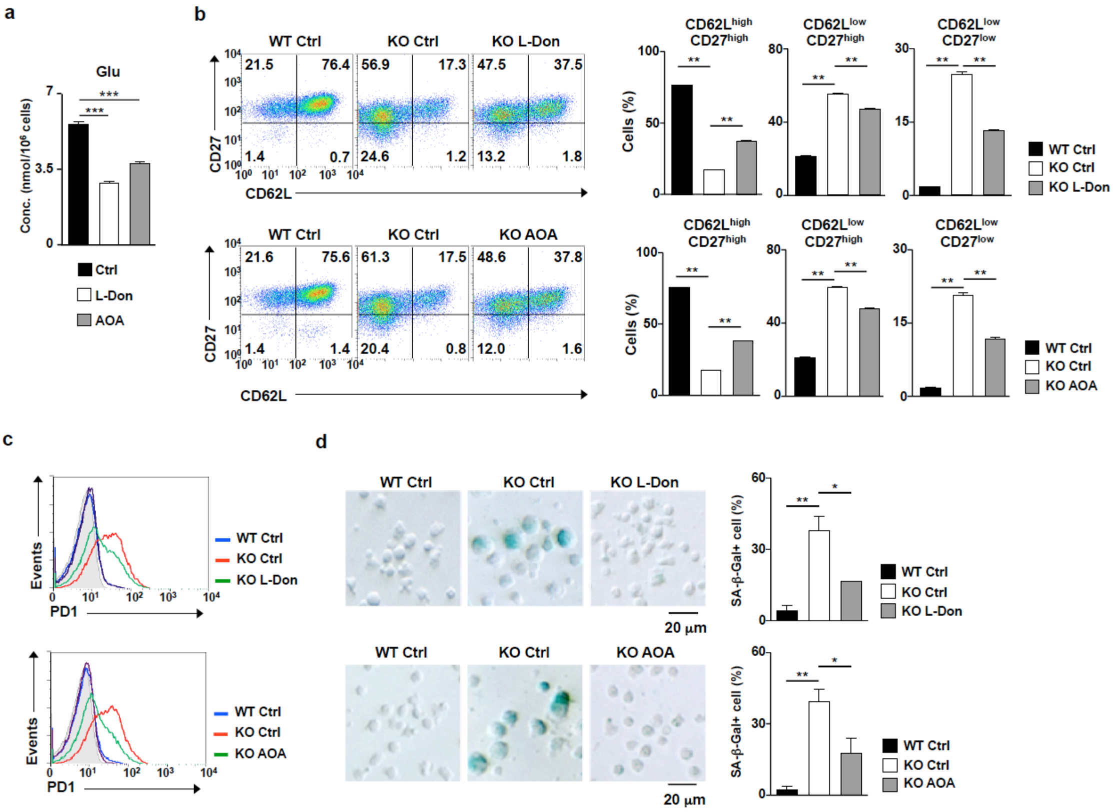

Supplementary Figure 11. The pharmacological inhibition of the glutamine metabolism partially restored the phenotype of *menin* KO CD8 T cells. (a) The intracellular levels of glutamate in the naïve CD8 T cells stimulated with anti-TCR-β mAb plus anti-CD28 mAb in the presence of IL-2 with or without the indicated inhibitor for 24 h are shown with the standard deviation (n = 3: biological replicates). **(b)** *Menin* KO naïve CD8 T cells were stimulated with anti-TCR-β mAb plus anti-CD28 mAb in the presence of IL-2 with or without an inhibitor (L-Don [1 μM] or AOA [1 mM]) for 2 days, and then the cells were further expanded with IL-2 in the absence of inhibitors for an additional 5 days. Representative staining profile of CD62L/CD27 (left) and the percentages with the standard deviation of $CD62L^{high}/CD27^{high}$, $CD62L^{low}/CD27^{high}$ and $CD62L^{low}/CD27^{low}$ cells in three independent cultures (right) are shown. **(c)** Representative FACS profiles of PD-1 on the cells in (b) are shown. **(d)** Representative pattern of SA β-Gal staining (left) and the percentages with the standard deviation of SA β-Gal positive cells on day 12 in the three independent cultures (right) are shown. (a) ***p<0.001 (Welch *t*-test). (b), (d) *p<0.05, **p<0.01 (Student's *t*-test).

Supplementary Figure 12

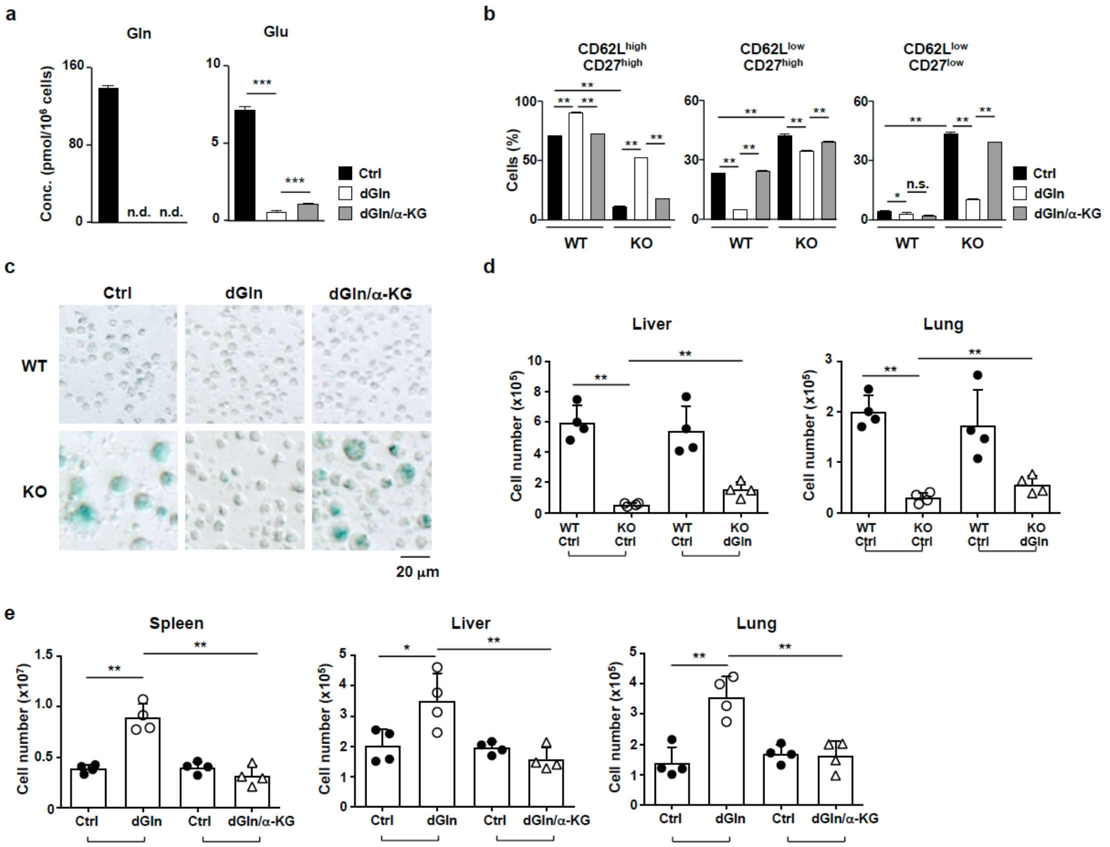

Supplementary Figure 12. The effect of glutamine-deprivation and α-KG on the phenotype of *menin* KO CD8 T cells. (a) The intracellular levels of glutamine and glutamate in the activated CD8 T cells under normal (Ctrl), glutamine-deprived (dGln), and glutamine-deprived supplemented with DM-α-KG (dGln/α-KG) conditions are indicated (n = 3: biological replicates). The results are presented with the standard deviation. WT naïve CD8 T cells were stimulated with anti-TCR-β mAb plus anti-CD28 mAb in the presence of IL-2 under the indicated conditions for 36 h. **(b)** The percentages of $CD62L^{high}/CD27^{high}$, $CD62L^{low}/CD27^{high}$ and $CD62L^{low}/CD27^{low}$ CD8 T cells in Figure 5a in three independent cultures with the standard deviation are shown. **(c)** A representative pattern of SA β-Gal staining of the cells in Figure 5e. **(d)** The absolute number of donor cells in the liver and lungs of mice in Figure 5f is indicated (mean ± SD, n = 4 per group: biological replicates). **(e)** Naïve CD8 T cells from WT OT1 Tg mice cultured under normal conditions ($Thy1.1^+$) or the indicated conditions ($Thy1.2^+$) were mixed at a 1:1 ratio and adoptively transferred into naïve C57/BL6 mice ($Thy1.1^+$ x $Thy1.2^+$). Twenty days after the adoptive transfer, the mice were infected

with *Lm*-OVA to activate the donor cells, and the number of OT1 Tg CD8 T cells was assessed. The donor cells from the spleen, liver, and lungs were analyzed on day 5 after the infection and the absolute number of donor cells in the spleen, liver and lungs (mean ± SD, n = 4 per group) is shown. (a) **$p<0.01$ (Welch's *t*-test), (b), (d), (e) *$p<0.05$, **$p<0.01$ (Student's *t*-test).

Supplementary Figure 13

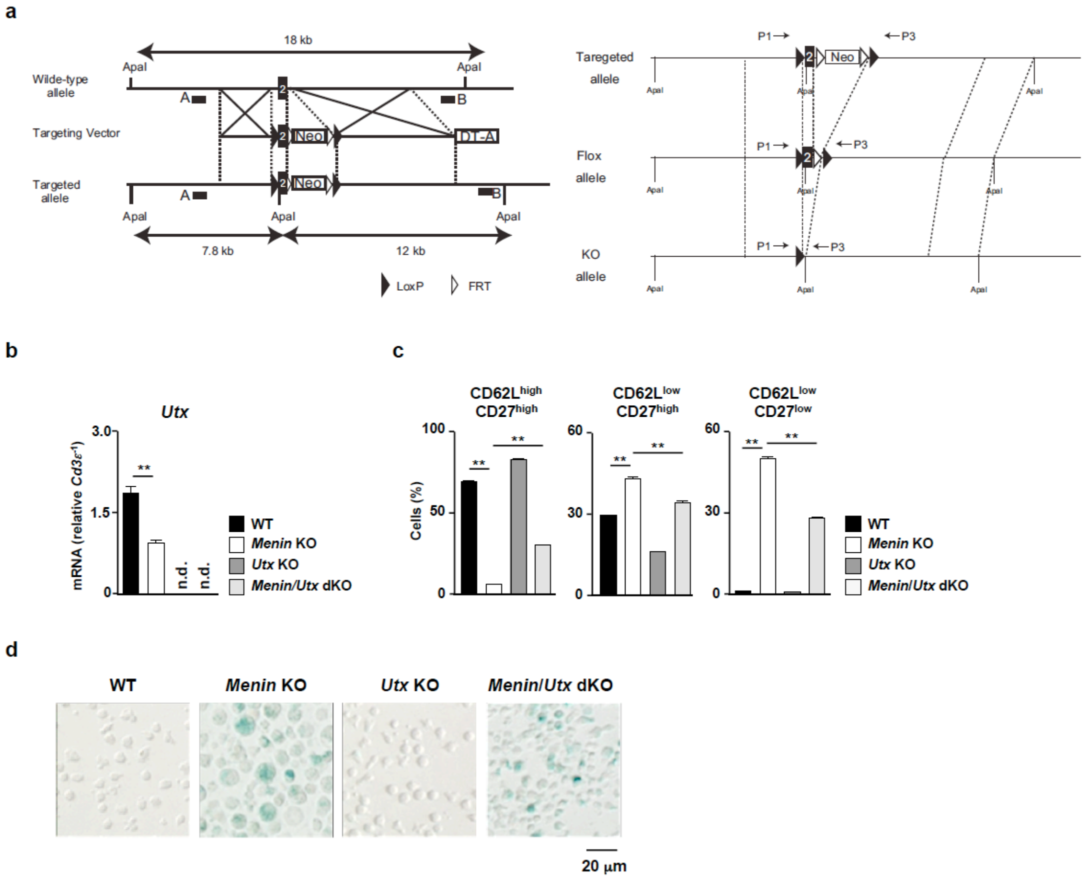

Supplementary Figure 13. Effect of utx deficiency on the induction of senescence in *menin* KO CD8 T cells. (a) A schematic diagram of the targeting construct for the *utx* gene and *utx*-floxed allele. The *utx*-floxed mice were crossed with CD4 Cre TG mice to generate T cell-specific *utx*-deficient mice. **(b)** The results of the quantitative RT-PCR analysis of *utx* in WT, *menin* KO, *utx* KO and *menin*/*utx* double KO effector CD8 T cells on day 7 after the initial TCR stimulation. The results are presented relative to the mRNA expression of *Cd3ε* with the standard deviations (n = 3: technical replicates). **(c)** Naïve CD8 T cells with the indicated genetic background were stimulated with anti-TCR-β mAb plus anti-CD28 mAb in the presence of IL-2 for 2 days and then further expanded with IL-2 for an additional 5 days. The percentages with the standard deviation of CD62L^{high}/CD27^{high}, CD62L^{low}/CD27^{high} and CD62L^{low}/CD27^{low} CD8 T cells in the three independent cultures are shown. **(d)** Representative patterns of SA β-Gal staining of CD8 T cells on day 12. **p<0.01 (Student's *t*-test).

Supplementary Figure 14

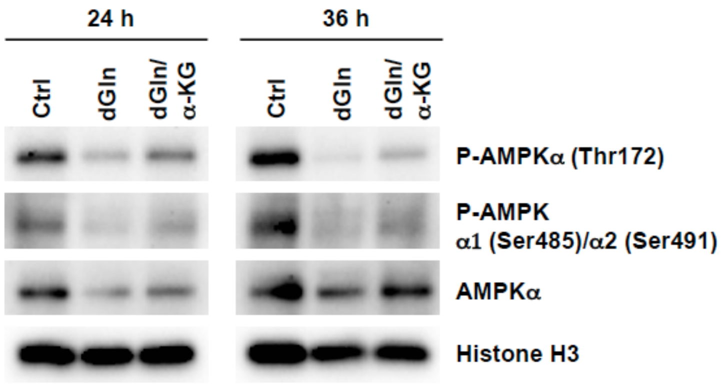

Supplementary Figure 14. Decreased phosphorylated and total AMPKα in activated CD8 T cells cultured under glutamine-deprived conditions. (a) The results of the immunoblot analysis of phospho-AMPKα (Thr172), phospho-AMPKα1 (Ser485), phospho-AMPKα2 (Ser491), AMPKα and histone H3 in CD8 T cells cultured under the indicated conditions for 24 h (**left**) or 36 h (**right**). The protein amount of histone H3 was used as a loading control.

Supplementary Figure 15

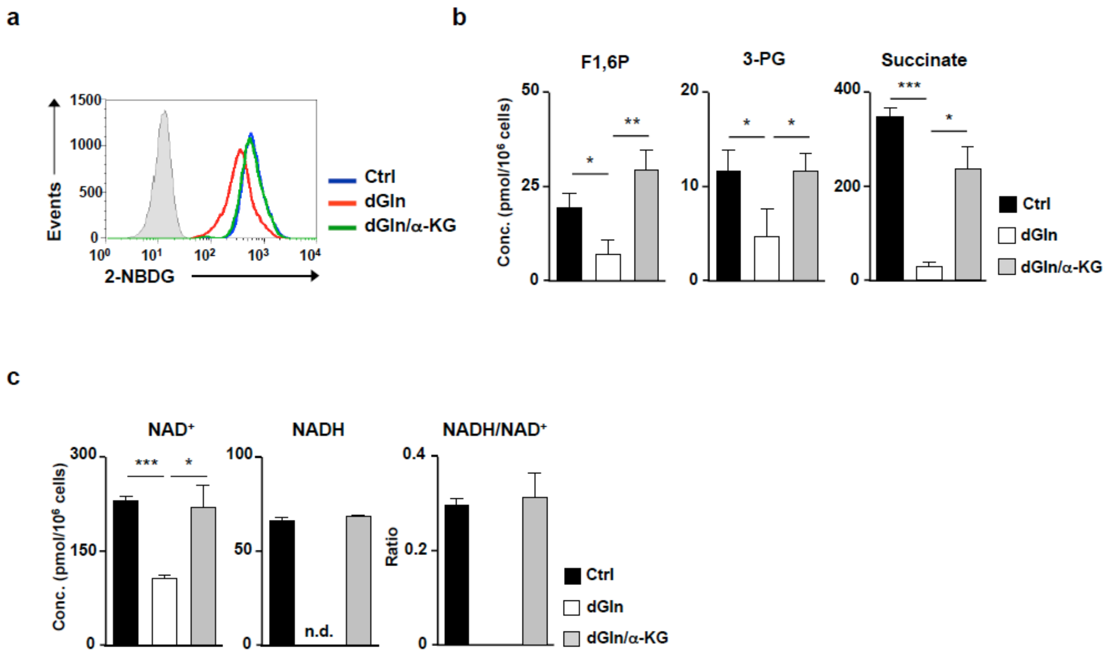

Supplementary Figure 15. The α-KG-dependent activation of glycolysis and glutaminolysis. (a) The incorporation of 2-NBDG in CD8 T cells cultured under the indicated conditions for 48 h is shown. **(b)** The intracellular amounts of fructose 1,6-diphosphate (F1, 6P), glycerate 3-phosphate (3-PG) and succinate in the activated CD8 T cells under normal (Ctrl), glutamine-deprived (dGln) and glutamine-deprived supplemented with dimethyl-α-KG (dGln/α-KG) conditions are indicated (n = 3; biological replicates). The results are presented with the standard deviation. WT naïve CD8 T cells were stimulated with anti-TCR-β mAb plus anti-CD28 mAb in the presence of IL-2 under the indicated conditions for 36 h **(c)** The intracellular amount of NAD^+ and NADH in CD8 T cells cultured under the indicated conditions as described in (b). NADH/NAD^+ is also indicated. *p<0.05, **p<0.01, ***p<0.001 (Welch's *t*-test).

Supplementary Figure 16

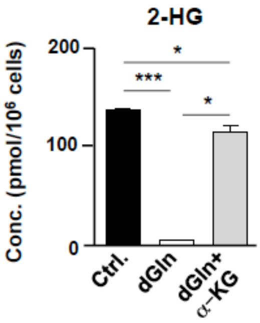

Supplementary Figure 16. The glutamine-α-KG axis controls intracellular concentration of 2-HG. The intracellular amounts of 2-hydroxyglutarate (2-HG) in the activated CD8 T cells under normal (Ctrl), glutamine-deprived (dGln) and glutamine-deprived supplemented with dimethyl-α-KG (dGln/α-KG) conditions are indicated (n = 3; biological replicates). The results are presented with the standard deviation. *p<0.05, ***p<0.001 (Welch's *t*-test).

Supplementary Figure 17

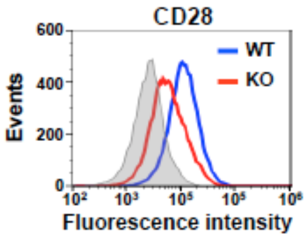

Supplementary Figure 17. Decreased expression of CD28 in *menin* KO CD8 T cells. A representative staining profile of CD28 on the cell surface of the WT and *menin* KO effector CD8 T cells. Naïve CD8 T cells from the spleen of the WT and *menin* KO mice were stimulated with anti-TCR-β mAb plus anti-CD28 mAb in the presence of IL-2 for 2 days. The cells were then further expanded with IL-2 for 5 days.

Supplementary Figure 18

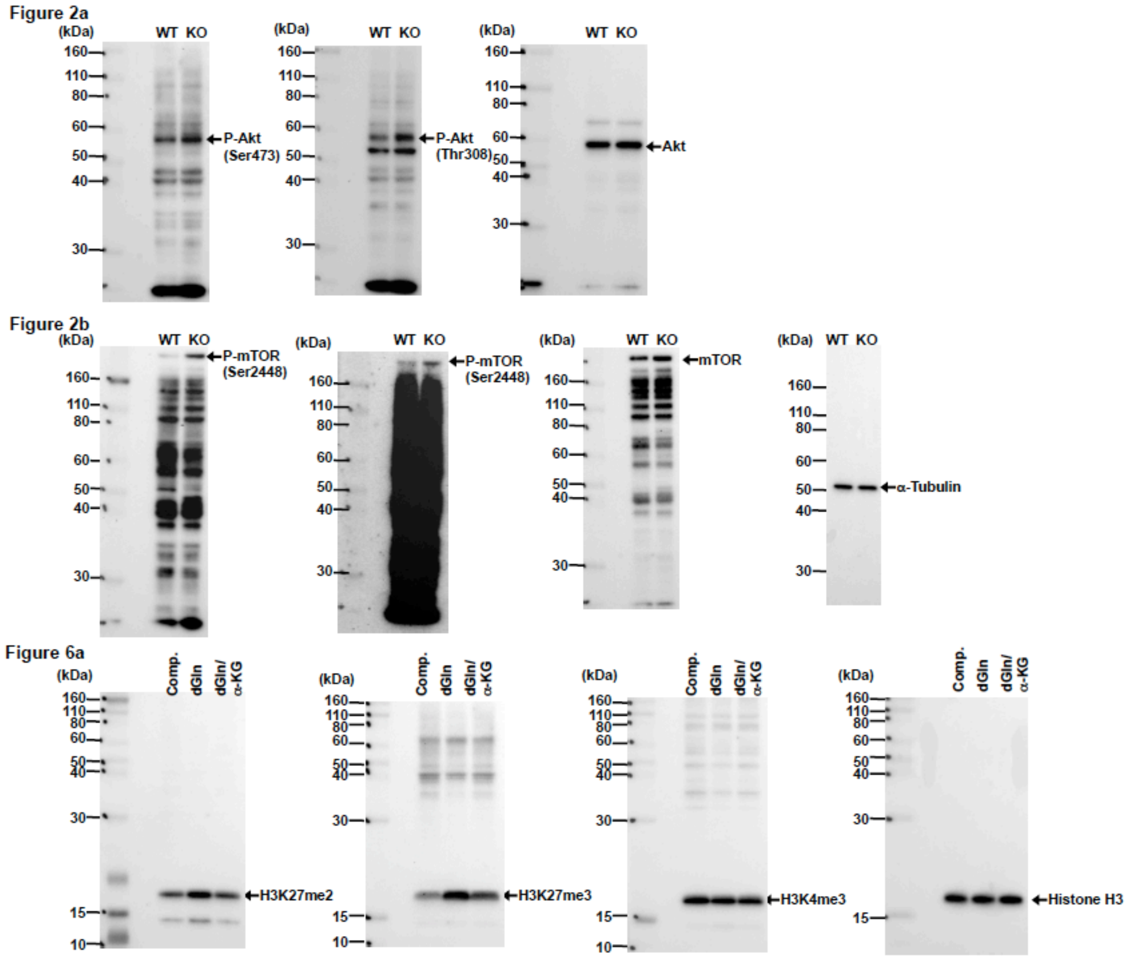

Supplementary Figure 18. Full size immunoblots of cropped blots in the main manuscript figures. Full size immunoblots of cropped blots for **Fig. 2a**, **Fig 2b** and **Fig 6a**.

Supplementary Figure 19

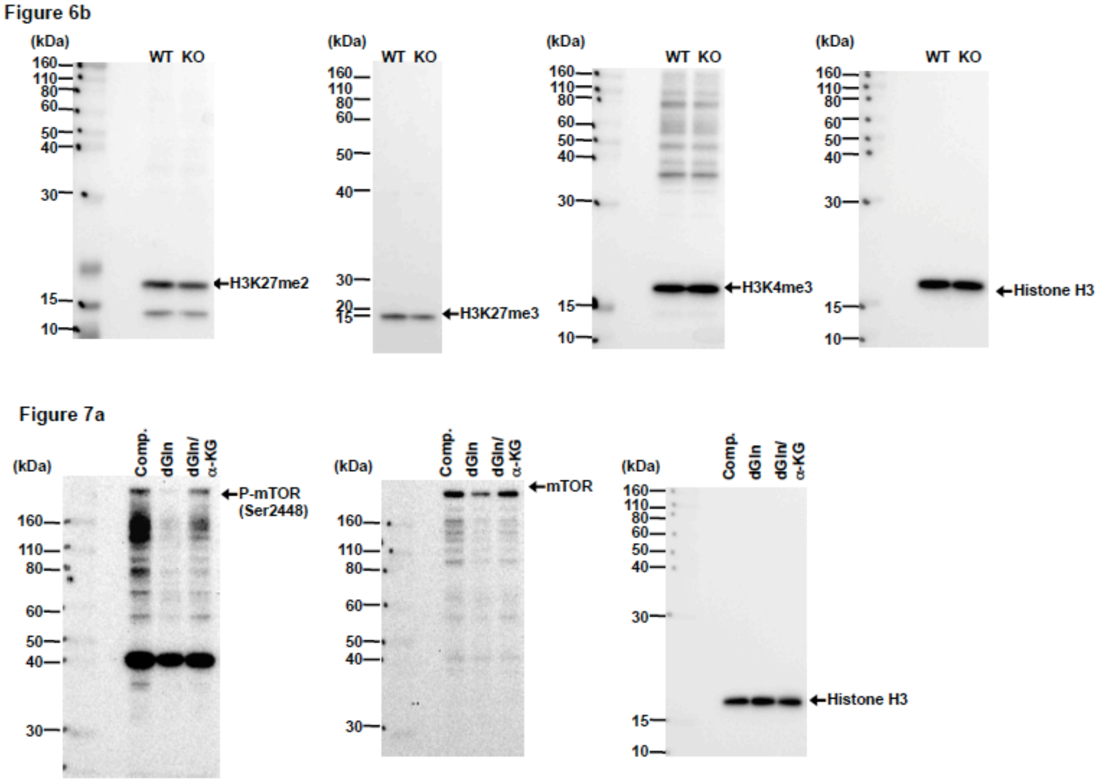

Supplementary Figure 19. Full size immunoblots of cropped blots in the main manuscript figures. Full size immunoblots of cropped blots for **Fig. 6b** and **Fig. 7a**.

Supplementary Figure 20

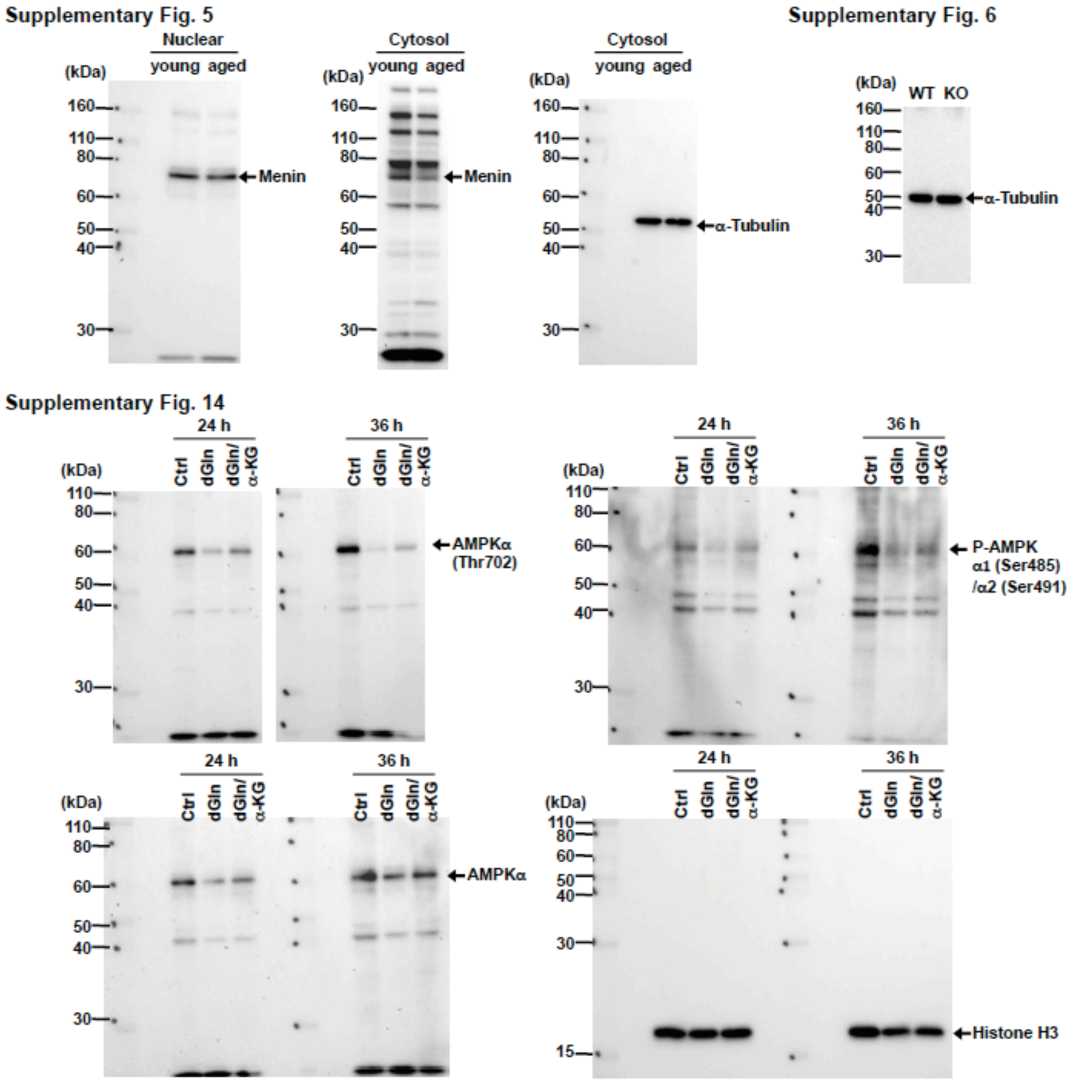

Supplementary Figure 20. Full size immunoblots of cropped blots in the main manuscript figures. Full size immunoblots of cropped blots for Supplementary **Fig. 5, Supplementary Fig. 6** and **Supplementary Fig. 14**.
